# Supplementary material for: Focused ultrasound enables selective actuation and Newton-level force output of untethered soft robots
Source: Nat Commun. 2024 Jun 18;15:5197. doi: 10.1038/s41467-024-49148-6 (PMC11189400; doi:10.1038/s41467-024-49148-6)
Supplement: Supplementary file 1 — Supplementary Information [file 41467_2024_49148_MOESM1_ESM.pdf]

Supplemental Materials

Focused Ultrasound Enables Selective Actuation and Newton-level Force Output of Untethered Soft Robots

Bo HAO[1†], Xin WANG[1†], Yue DONG[2†,*], Mengmeng SUN[1], Chen XIN[1], Haojin YANG[1], Yanfei CAO[1], Jiaqi ZHU[1], Xurui LIU[1], Chong ZHANG[1], Lin SU[1], Bing LI [2,*] and Li ZHANG[1,3,4,5,6,*]

[1]Department of Mechanical and Automation Engineering, The Chinese University of Hong Kong, Hong Kong, SAR 999077, P.R. China

[2]Guangdong Provincial Key Laboratory of Intelligent Morphing Mechanisms and Adaptive Robotics, School of Mechanical Engineering and Automation, Harbin Institute of Technology, Shenzhen 518055, China

[3]Multi-Scale Medical Robotics Center, Hong Kong Science Park, Shatin NT, Hong Kong, SAR 999077, P.R. China

[4]CUHK T Stone Robotics Institute, The Chinese University of Hong Kong, Hong Kong, SAR 999077, P.R. China

[5]Chow Yuk Ho Technology Center for Innovative Medicine, The Chinese University of Hong Kong, Hong Kong, SAR 999077, P.R. China

[6]Department of Surgery, The Chinese University of Hong Kong, Hong Kong, SAR 999077 P.R. China;

*Corresponding Author: lizhang@cuhk.edu.hk (L.Z.); libing.sgs@hit.edu.cn (B.L.); dongyue@hit.edu.cn (Y.D.)

†These authors contributed equally to this work.

Supplementary Sections

Supplementary Section 1. Acoustic pressure amplitude calibration

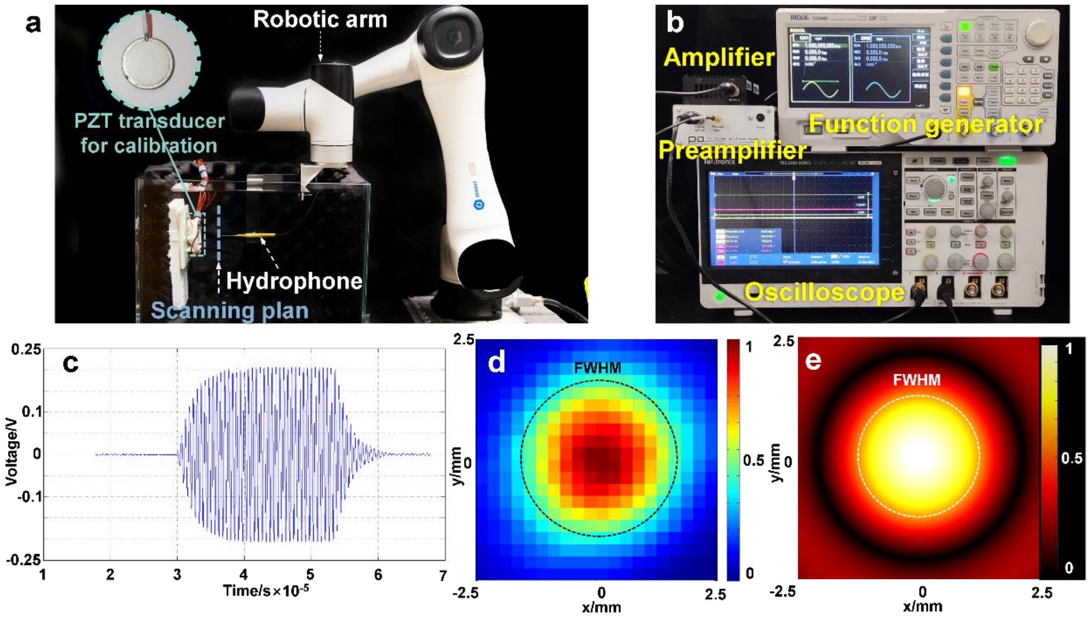

Supplementary Fig. 1: Acoustic pressure amplitude calibration. (a) Photograph of the PZT transducer and measurement system. (b) Signal generation and analysis system for pressure amplitude calibration. (c) The recorded signal of the hydrophone capturing the maximum pressure during the experiment. (d) The measured and (e) simulated normalized acoustic pressure generated by the PZT transducer at the scanning plane. FWHM represents the full width at half maximum.

To calibrate the acoustic pressure amplitude, a PZT transducer (20 mm diameter, 1.2 mm thickness) was placed in a water tank filled with deionized water after degassing. A needle hydrophone was mounted at the end of a robotic arm, which moved in a plane located 45 mm away from the transducer surface, as depicted in Supplementary Fig. 1a.

An electrical signal (1.7 MHz, 22.3 V_{pp}, 50 pulses) was generated by a function generator and amplified by a power amplifier. The hydrophone measurements were passed through a preamplifier to an oscilloscope and collected by a PC, as shown in Supplementary Fig. 1b. The recorded signal captured the maximum pressure during the experiment and presented in Supplementary Fig. 1c. Considering that the hydrophone's sensitivity at 1.7 MHz was 1128 mV/MPa, the maximum pressure reached approximately 182 kPa, indicating a pressure amplitude constant of 8.16 kPa/V_{pp}. (peak-to-peak voltage abbreviated as V_{pp})

Supplementary Fig. 1d and 1e illustrates the measured and simulated normalized pressure profiles at the scanning plane, respectively. The simulated half-amplitude region diameter (2.5 mm) exhibits small deviation from the experimental results (3 mm). This discrepancy might be attributed to the influence of the hydrophone within the acoustic field or potential measurement errors.

Supplementary Section 2. Sound speed measurement and acoustic impedance calculations

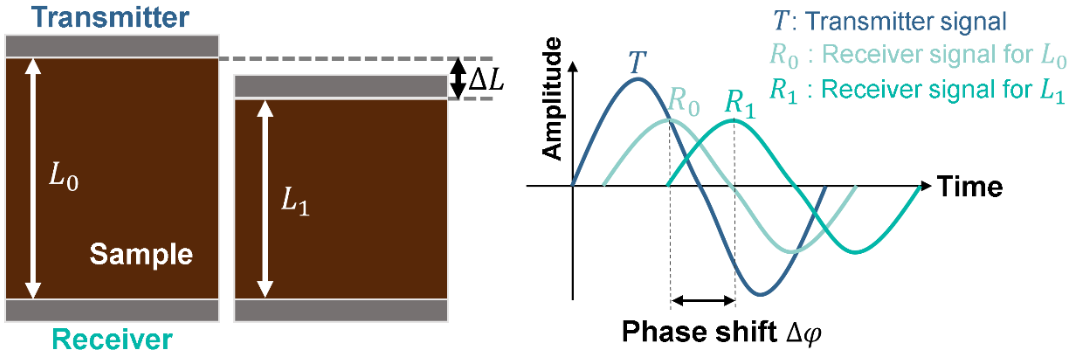

Supplementary Fig. 2: Schematic illustration showing the measurement of sound speed.

As shown in Supplementary Fig. 2, the signal generator produces a continuous sinusoid wave (that matches with the resonant frequency f of the transducer), which drives the transmitter to generate a mechanical vibration that travels through the material between two transducers and thus converted into electrical signal by the receiver. By reducing the distance of the sample between the transmitter and receiver (ΔL), the phase difference between transmitted signal and received signal will shift by $\Delta\varphi$, thus the sound speed (v) in the sample can be calculated as follows:

$$v = 2\pi f \frac{\Delta L}{\Delta\varphi} \tag{1}$$

The measured density and sound speed for Ecoflex 00-30 and Fe_3O_4NPs doped Ecoflex 00-30 can be found in Supplementary Table 1.

Supplementary Table 1: Acoustic impedance of different materials.

Material	Density (10^3 kg m^{-3})	Velocity (m s^{-1})	Impedance (MRayl)
Water	1	1480	1.48
Soft tissue[1]	1.1	1540	1.69
Fat[1]	0.95	1450	1.38
Ecoflex 00-30	1.061±0.005	1035±14	1.10
Fe_3O_4NPs doped Ecoflex 00-30	1.10±0.003	1074±21	1.18

Supplementary Section 3. Ultrasound-induced temperature and pressure elevation measurement

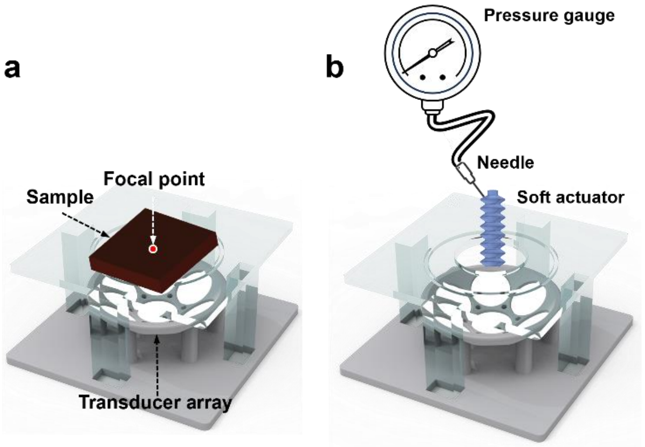

Supplementary Fig. 3: Schematic illustration showing the temperature and pressure measurement method. (a) Schematic illustration of the experimental setup for ultrasound-induced heating measurement and (b) pressure measurement inside the soft actuator.

To investigate the influencing factors of the acoustothermal effect, sample films with a thickness of 2 mm are positioned in the plane that passes through the acoustic focal point of the transducer array (as shown in Supplementary Fig. 3a). An infrared camera (FLIR ONE, Teledyne FLIR) is used to record the temperature change during the heating process. For pressure measurement, a typical soft actuator (referred to as the “elongation actuator” in Fig. 3c) is aligned with the acoustic field, and the internal pressure changes are measured using a pressure gauge (as shown in Supplementary Fig. 3b).

Supplementary Section 4. Acoustic attenuation measurement and discussion

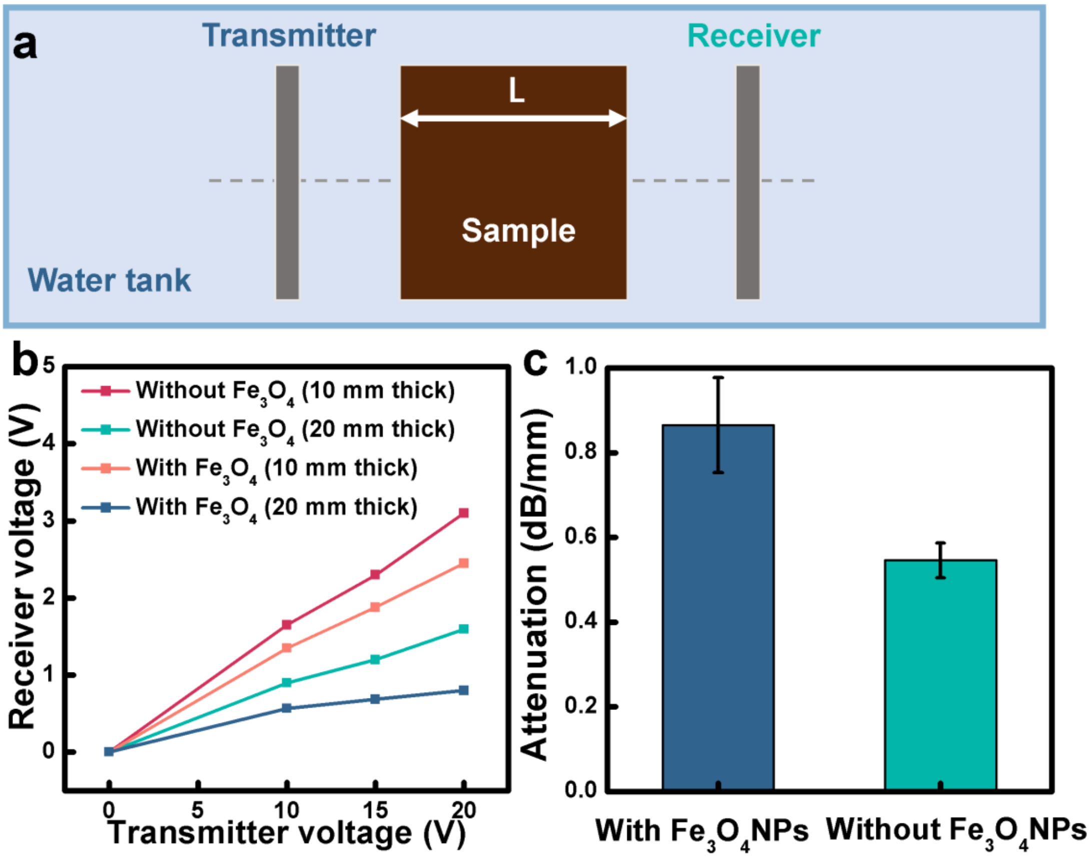

Supplementary Fig. 4: Acoustic attenuation measurement. (a) Schematic illustration of the measurement of acoustic attenuation. (b) Relationship between received and transmitted voltage for different samples. (c) The calculated acoustic attenuation of Ecoflex 00-30 and Fe_3O_4NPs doped Ecoflex 00-30.

Supplementary Fig. 4 illustrates the measurement of acoustic attenuation. As shown in Supplementary Fig. 4a, a continuous sinusoidal wave is generated by a signal generator at the resonant frequency (*f*) of the transducer. This wave drives the transmitter, which produces mechanical vibrations that pass through the sample and is converted into electrical signals by the receiver. The voltages of the receiver and transmitter are recorded to calculate the ratio of their signal amplitudes (Supplementary Fig. 4b). The voltage ratio of the receiver to the transmitter decreases with the thickness increase of the sample film. Additionally, the doping of Fe_3O_4NPs also decreases this voltage ratio.

The acoustic attenuation (α) of the Ecoflex 00-30 film with and without Fe_3O_4NPs doping at 1.7 MHz can be calculated using the following equation:

$$\alpha = 20log\left(\frac{P_1}{P_2}\right)\frac{1}{\Delta L} \tag{2}$$

Here, P_1 and P_2 are the received pressure amplitude of samples with thicknesses L_1 and L_2, respectively. ΔL ($\Delta L =$ L_1 - L_2) represents the thickness difference between L_1 and L_2. The pressure amplitude ratio (P_1/P_2) of the Ecoflex 00-30 samples (L_1 = 10 mm and L_2 = 20 mm) and Fe_3O_4NPs doped Ecoflex 00-30 samples are 1.88 ± 0.09 and 2.72 ± 0.35, respectively. Consequently, the calculated acoustic attenuation coefficient for Ecoflex 00-30 and Fe_3O_4NPs doped Ecoflex 00-30 are 0.54 dB/mm and 0.84 dB/mm (Supplementary Fig. 4c), respectively.

When the actuators are actuated by the acoustic field, the heat source Q of the material heated can be calculated as [2]:

$$Q = 2\alpha I = \frac{\alpha\left(abs(P)\right)^2}{Z} \tag{3}$$

where I is the magnitude of the acoustic intensity, P is the acoustic pressure, and Z is the acoustic impedance. Based on the measured parameters, the introduction of Fe_3O_4NPs into the polymer increases heat absorption under identical acoustic fields. Combining this with the heat transfer equation, the temperature field distribution can be obtained using equation (4):

$$\rho C_p \frac{\partial T}{\partial t} = \nabla \cdot (\kappa \nabla T) + Q \tag{4}$$

where T represents the temperature field, ρ is the material density, C_p is the specific heat of the material, and κ is the thermal conductivity.

At the initial stage of heating, the temperature of the material is close to the environment, heat dissipation due to the gradient is negligible (as shown in equation (4), $\nabla \cdot (\kappa \nabla T) \approx 0$). Therefore, most of the heat that induces the temperature change

in the actuator originates from the acoustothermal effect. Moreover, since the attenuation of the Fe_3O_4NPs doped Ecoflex 00-30 is more considerable, more heat is generated, resulting in a faster temperature rise (Fig. 2d). Similarly, when the material reaches the steady-state temperature, most of the heat produced by the acoustothermal effect is used to balance the heat dissipation, causing the slope of the temperature curve to decrease until a steady state is reached. Furthermore, a larger input heat (Q) caused by a larger voltage induces a stronger acoustothermal effect, leading a higher steady-state temperature (Equation (4) and Fig. 2e).

Supplementary Section 5. Force measurement of soft actuator for biopsy and tissue patching

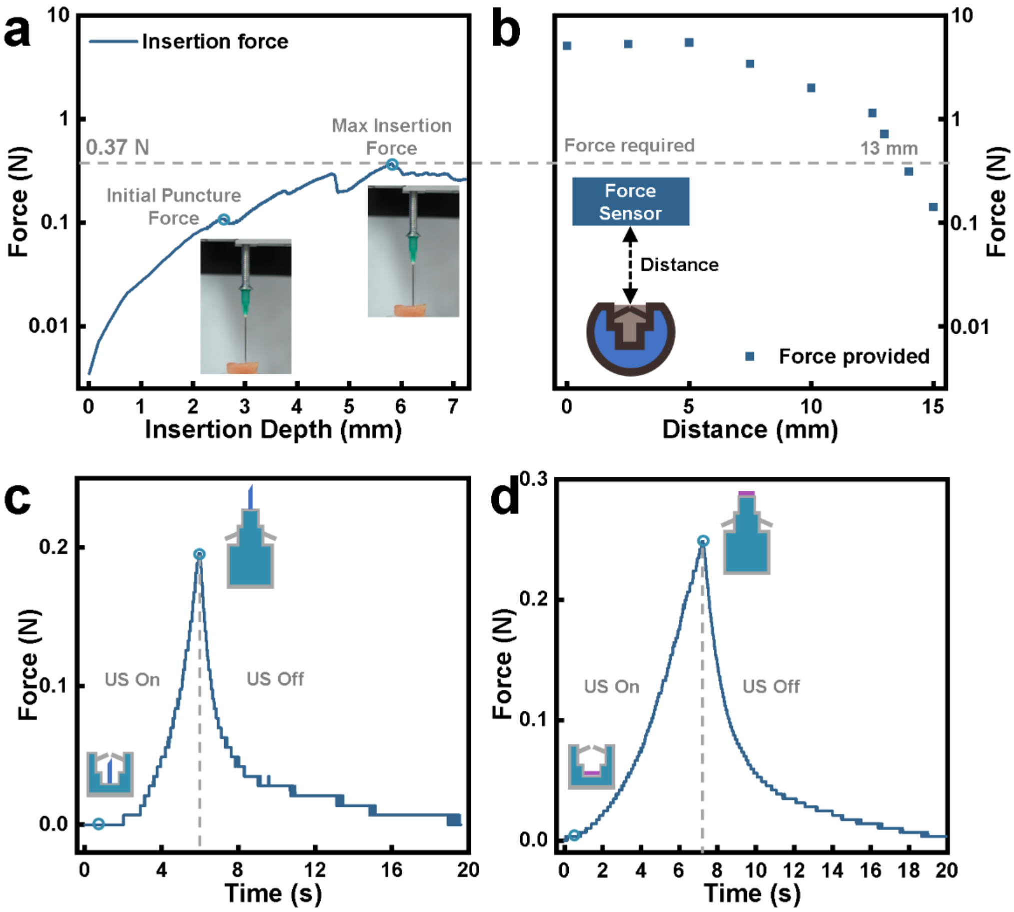

Supplementary Fig. 5: Force measurement. (a) The detected force during the process of needle inserts into tissue. The maximum value of the insertion force is 0.37 N. (b) The relationship between actuation force and the distance from the actuator to the target. (c) The force change during (c) needle insertion and retrieval process and (d) the tissue patching process.

To verify whether the actuation force of the soft actuator is sufficient for biopsy, the total needle insertion force is measured during the insertion procedure, as shown in Supplementary Fig. 5a. The maximum needed insertion force is approximately 0.37 N, which is lower than the output force of the soft actuator when it is less than 13 mm away from the target (Supplementary Fig. 5b). This indicates that the actuator driven by FUPT is well-suited for tissue acquisition tasks. Furthermore, we have recorded the force during biopsy and tissue patching procedures as shown in Supplementary Fig. 5c and 5d, demonstrating that the soft actuator can be actuated within a few seconds and generate a sufficiently large mechanical output to complete tasks.

Supplementary Section 6. Influence factors of cooling time for the soft actuator

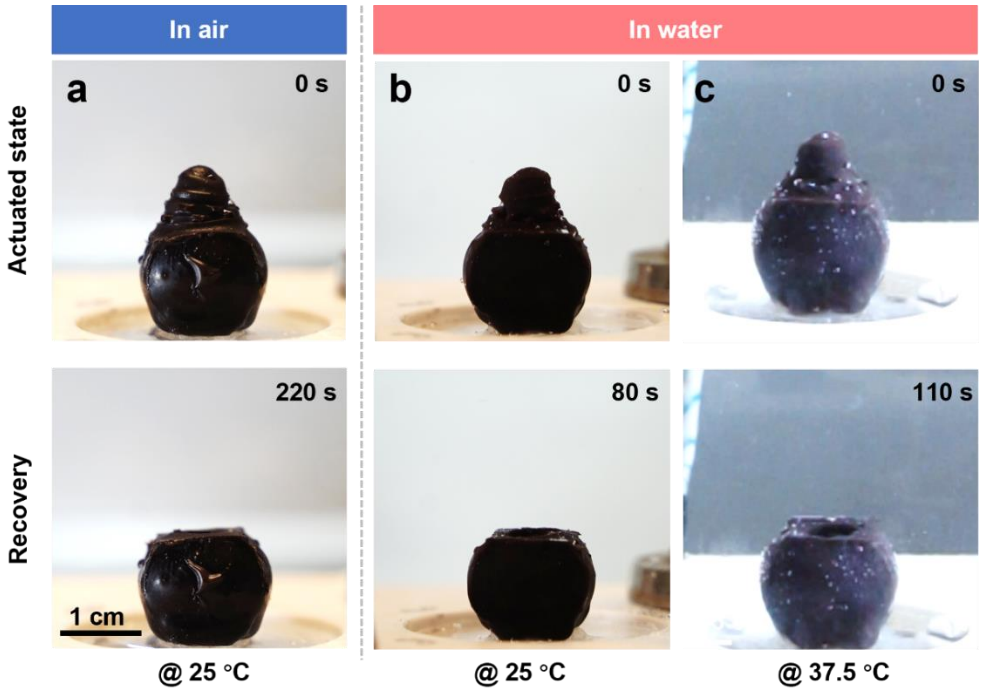

Supplementary Fig. 6: Comparison of recovery time (a) in air, 25 °C, (b) in water, 25 °C, and (c) in water 37.5 °C.

The timeframe of the recovery phase is a crucial aspect to consider when evaluating the applicability of the presented soft robot for time-sensitive applications. In our experiments, the recovery time required for the biopsy and patching soft robot ranging from approximately 80 s to 220 s. The cooling speed of the soft actuator is influenced by several factors, including the temperature difference between the actuator and the environment, the thermal conductivity of the materials used, and the structural geometry. For example, the cooling time can be significantly reduced to approximately 80 s when submerged in water (Supplementary Fig. 6b), compared to approximately 220 sin air at 25 °C (Supplementary Fig. 6a). This observation can be attributed to the higher thermal conductivity of water, which enhances the heat dissipation process and accelerates the cooling speed. Furthermore, the larger the temperature difference between the environment and the soft robot, the faster the thermal conduction and the shorter the cooling time. For instance, the soft actuator was cooled down to unactuated state within 80 s when immersed in 25 °C water, while the time is extended to 110 s in 37.5 °C (Supplementary Fig. 6c).

The thermal conductivity capability of the materials embedded within the soft robot also plays a role in the cooling speed. For instance, the inclusion of Fe_3O_4NPs in the structure improves the thermal conduction, enabling faster cooling rates. Additionally, the size of the actuator has a direct impact on the heating and cooling time. According to the scaling law, smaller actuators exhibit faster response rates. This is because the amount of heat required for actuation is proportional to the volume, which scales with the cube of the characteristic size. In contrast, the heat dissipation area is proportional to the square of the characteristic size. Therefore, smaller actuators dissipate heat more efficiently and experience faster heating and cooling rates. Our experimental results confirm this trend, as the 4 mm-sized actuator (Fig. 4h) demonstrated significantly shorter actuation and recovery times (within several seconds).

To summarize, the recovery phase of the soft robot can be adjusted by considering several factors. Immersion in a liquid environment, such as water, accelerates the cooling process, while the choice of materials, thickness of the structure, and size of the actuator also influence the heating and cooling rates. By optimizing these parameters, the soft robot can be tailored to meet the requirements of time-sensitive applications.

Supplementary Section 7. Permeability evaluation of the phase transition material

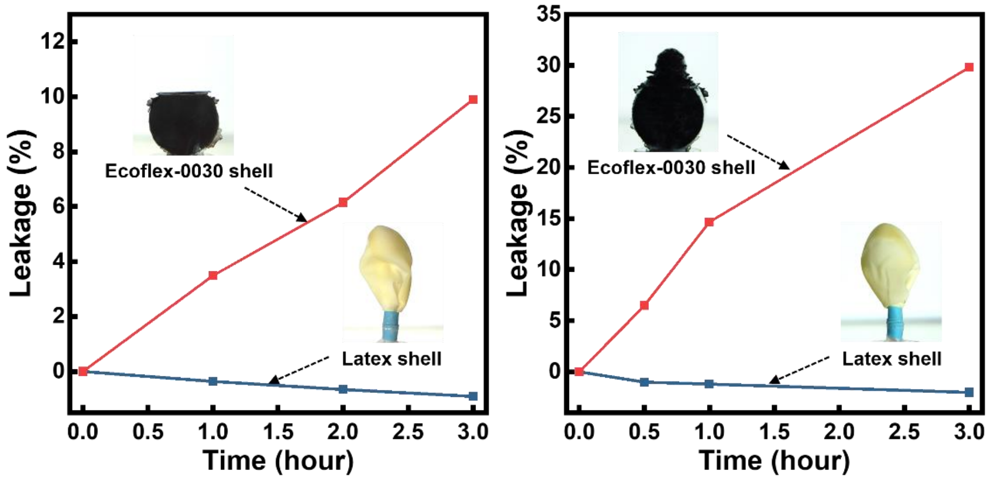

Supplementary Fig. 7: Leakage measurement of the phase-transition material inside the soft actuator over time. (a) In unactuated state (37 °C) and (b) in actuated state (45 °C).

To examinate the leakage of the internal phase transition material of the actuator, we injected approximately 1.1 g of Novec 7000 into an empty actuator and measured its mass after each test to estimate the leakage rate. Considering the application scenarios, we submerged the actuator in water at body temperature (i.e. ~ 37 °C). As shown in Supplementary Fig. 7, the mass of the actuator gradually decreased over time in the 37 °C environment, indicating the present of internal material leakage. Within the 3 h testing duration, we observed a leakage of approximately 0.1 g (around 10% of the total phase transition liquid) with an average leakage rate of approximately 3.3% per hour. After 16 h, the leakage of the enclosed liquid approximately 0.28 g (~ 25. 5%), indicating a slower leakage rate compared to the initial state. It is important to note that our designed robot and actuators are intended for short-term operation rather than long-term implantation, so the amount of leakage within a relatively short period is negligible, as verified in Supplementary Fig. 8, where after 20 cycles, the actuator is still able to meet the design objective of extruding the internal component.

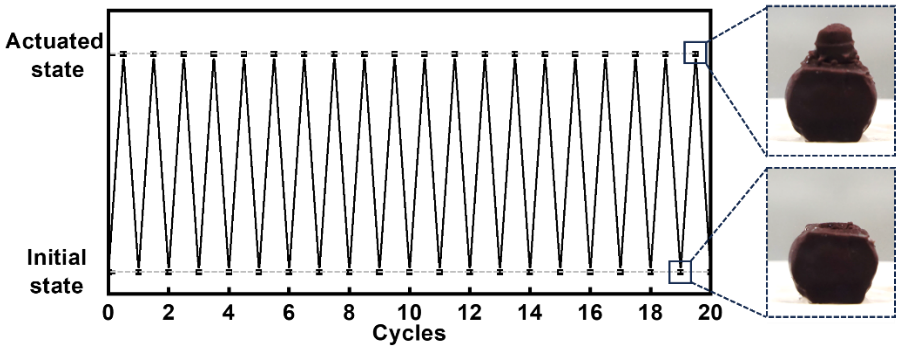

Supplementary Fig. 8: The repeatable actuation performance (i.e. 20 cycles) of the soft actuator.

To address the issue of gas leakage during heating, the materials with better gas tightness can be considered. As shown in Supplementary Fig. 7, the shell material was replaced from Ecoflex 00-30 (approximately 1 mm thick) to latex (approximately 0.1 mm thick), resulting in no significant leakage observed at both 37 °C and 45 °C (i.e. actuated state). Therefore, if the FUPT-based soft robotics are to be extended for long-term implanted devices actuation, it would be necessary to replace or modify the materials to enhance their sealing properties.

Supplementary Section 8. Fabrication of the soft actuators/robots

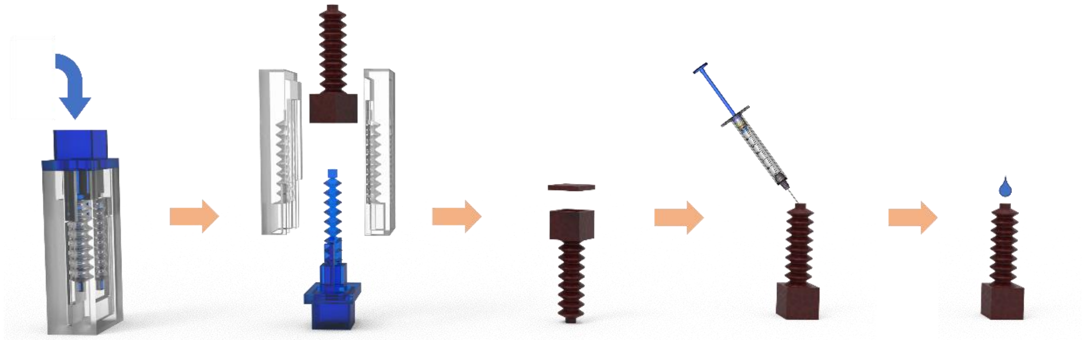

Supplementary Fig. 9: Fabrication of the phase transition soft actuators.

The phase transition soft actuators were fabricated by template method. Specifically, as shown in Supplementary Fig. 9, the mold was designed based on the structures of the actuator and then 3D printed by liquid crystal display (LCD) 3D printer (LD-002H, Shenzhen Creality 3D Technology Co., Ltd.). The surface treatment was indispensable for the printed molds so that the silicone polymerization process can be entirely complete.[3] In a typical experiment, the printed mold post-cured in 405 nm LED UV box for 30 mins, followed by treating with oxygen plasma for 20 mins. Next, 1H,1H,2H,2H-perfluorooctyl trichlorosilane (PFTS) were vapor-deposited on the mold surface in a vacuum desiccator for 8 h (100 °C). Finally, after washing by ethyl alcohol 3 times and dried at 40 °C for 3 h, the mold with silanized surface was successfully fabricated.

For the fabrication of the soft actuators, the component A and B of silicone rubber (Ecoflex 00-30, Smooth-On Inc.) with 1:1 volume ratio were stirred for 3 min, then the Fe_3O_4NPs (20 nm average diameter, Sigma-Aldrich Company) were added followed by stirring until thoroughly mixing and degassing in a vacuum chamber. Next, the above-mentioned mixed polymer elastomer was poured into treated mold and degassed for 10 mins. The mold filled with precursor was then placed in oven (60 °C) for 2 h until platinum catalyzed polymerization process finished (step 1-2 in Supplementary Fig. 9). After demolding and sealing process, the engineering liquid (Novec 7000) was

injected via syringe (step 2-4 in Supplementary Fig. 9). Finally, the soft actuator with the hollow structure were successfully fabricated after resealing. In the current study, the mass ratio of Fe_3O_4NPs in the precursor was 5 wt%.

Supplementary Section 9. Material mechanical property for finite element method

The finite element method was applied to predict the deformation and motion of the actuators/robots[4]. Specifically, according to the continuum mechanics, the deformation of a solid can be described:[5]

$$\mathbf{x} = \mathbf{x}(\mathbf{X}, t) = \mathbf{X} + \mathbf{u}(\mathbf{X}, t) \tag{5}$$

where the coordinates $\mathbf{X}$ and $\mathbf{x}$ are denoted as the locations of the material particle before and after deformation, respectively. $\mathbf{u}(\mathbf{X}, t)$ is the displacement vector pointing from $\mathbf{X}$ to $\mathbf{x}$.

The deformation gradient is given by:

$$\mathbf{x} = \mathbf{x}(\mathbf{X}, t) = \mathbf{X} + \mathbf{u}(\mathbf{X}, t) \tag{6}$$

$$\mathbf{F} = \mathbf{I} + \nabla\mathbf{u} = \begin{bmatrix} 1 + \frac{\partial u}{\partial X} & \frac{\partial u}{\partial Y} & \frac{\partial u}{\partial Z} \\ \frac{\partial v}{\partial X} & 1 + \frac{\partial v}{\partial Y} & \frac{\partial v}{\partial Z} \\ \frac{\partial w}{\partial X} & \frac{\partial w}{\partial Y} & 1 + \frac{\partial w}{\partial Z} \end{bmatrix} \tag{7}$$

where $\mathbf{F}$ is the Jacobian matrix of the transformation, for incompressible hypothesis, the corresponding determinant $det(\mathbf{F}) = 1$, $\mathbf{I}$ is the identity matrix. By making use of the polar decomposition theorem, $\mathbf{F}$ can be decomposed into a product of a pure rotation matrix ($\mathbf{R}$) and a right stretch tensor ($\mathbf{U}$) given in the material frame:

$$\mathbf{F} = \mathbf{RU} \tag{8}$$

The right Cauchy-Green deformation tensor $\mathbf{C}$ is defined by:

$$\mathbf{C} = \mathbf{F}^{\mathbf{T}}\mathbf{F} = \mathbf{U}^{\mathbf{T}}\mathbf{R}^{\mathbf{T}}\mathbf{RU} = \mathbf{U}^{\mathbf{2}} \tag{9}$$

By subtracting the identity tensor from the right Cauchy-Green deformation tensor **C,** the Green-Lagrange strain tensor $\boldsymbol{\varepsilon}$ is defined:

$$\boldsymbol{\varepsilon} = \frac{1}{2}(\mathbf{C} - \mathbf{I}) = \frac{\mathbf{1}}{\mathbf{2}}(\mathbf{F}^{\mathbf{T}}\mathbf{F} - \mathbf{I}) \tag{10}$$

which can then be written on component form as:

$$\varepsilon_{ij} = \frac{1}{2}\left(\frac{\partial u_i}{\partial X_j} + \frac{\partial u_j}{\partial X_i} + \frac{\partial u_k}{\partial X_i}\frac{\partial u_k}{\partial X_j}\right) \tag{11}$$

For the strain tensor, we have three fundamental invariants:

$$\begin{cases} I_1(\varepsilon) = trace(\varepsilon) \\ I_2(\varepsilon) = \frac{1}{2}(trace(\varepsilon)^2 - trace(\varepsilon^2) \\ I_3(\varepsilon) = det(\varepsilon) \end{cases} \tag{12}$$

Using the principle stretches $\Lambda_i = \frac{l_i}{L_i}$, which is given by the ratio of deformed length (l_i) to undeformed length (L_i), the principal invariants reduce to:

$$I_1 = \Lambda_1^2 + \Lambda_2^2 + \Lambda_3^2, I_2 = \Lambda_1^2\Lambda_2^2 + \Lambda_2^2\Lambda_3^2 + \Lambda_1^2\Lambda_3^2, I_3 = \Lambda_1^2\Lambda_2^2\Lambda_3^2 \tag{13}$$

For incompressible material ($I_3 = 1$), the stress-stretch relations are obtained from the strain energy function by virtual work considerations:

$$\sigma = 2\left(\Lambda_1^2\frac{\partial W}{\partial I_1} - \Lambda_1^{-2}\frac{\partial W}{\partial I_2}\right) + p \tag{14}$$

where σ is the principal Cauchy stress, p is the hydrostatic pressure which can be determined from the equilibrium equations and boundary conditions, the strain energy density function W is the amount of energy stored elastically in the material under the state of stretch. For Ogden hyperelastic materials,[6,7] the stored energy of the compressible material is given in terms of principal stretches:

$$\begin{aligned} W &= \sum_{i=1}^{N} \frac{\mu_i}{\alpha_i}\left[\Lambda_1^{\alpha_i} + \Lambda_2^{\alpha_i} + \Lambda_3^{\alpha_i} - 3\right] + W_{\mathrm{vol}} \\ W_{\mathrm{vol}} &= f\left(\frac{1}{2}J^2 - J + \frac{1}{2}\right) - \sum_{i=1}^{N} \mu_i \ln J \end{aligned} \tag{15}$$

where $J = \Lambda_1\Lambda_2\Lambda_3 = 1$ for the material is incompressible, and μ_i, α_i, f are the material constants. The axial component of stress is given as:

$$T_{kk} = \frac{\Lambda_k}{J}\frac{\partial W}{\partial \Lambda_k} + \frac{\partial W}{\partial J};\ k = 1,2,3 \tag{16}$$

For the uniaxial tensile test, the axial stress in the compressible Ogden model is reduced to:

$$T_{11} = \sum_{i=1}^{N} \frac{\mu_i}{J}\left(\Lambda_1^{a_i} - J^{a_i/2}\Lambda_1^{-a_i/2}\right) \tag{17}$$

When an incompressible condition ($J = 1$) is imposed, the axial stress in the direction of the load is given by:

$$\sigma_1 = \sum_{i=1}^{N} \mu_i\left(\Lambda_1^{a_i} - \Lambda_1^{-a_i/2}\right) \tag{18}$$

where the stretch can also be replaced by the strain:

$$\Lambda_1 = 1 + \varepsilon_1 \tag{19}$$

Which gives the strain-stress relationship under axial stress condition, using a Levenberg-Marquardt solver, we have fitted the Ogden model with the experimental results (the order N of the Ogden model is selected to be 3), the fitted curves are provided in Supplementary Fig. 10 with the material parameters shown in Supplementary Table 2.

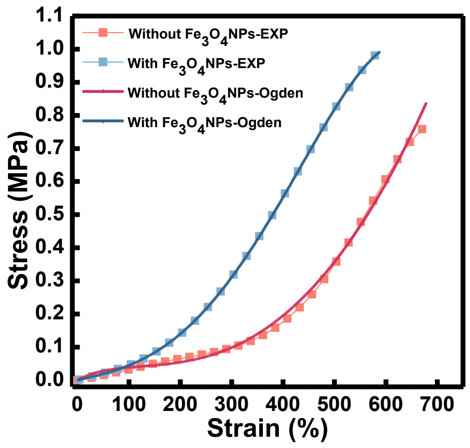

Supplementary Fig. 10: The stress-strain curves of Ecoflex 00-30 and Fe_3O_4NPs doped Ecoflex 00-30 films. Where the scatters were obtained from experiments (EXP) and the lines (Ogden) represent the fitted model.

Supplementary Table 2: Hyperelastic material property.

Parameters	Without Fe_3O_4NPs (Ecoflex 00-30)	With Fe_3O_4NPs
μ_1 (Pa)	-6.21E6	4692.13
a_1	1.82	3.47
μ_2 (Pa)	6.11E6	-96.35
a_2	1.83	5.03
μ_3 (Pa)	-6.63E7	-8860.58
a_3	-0.0026	-1.83

Supplementary Figures

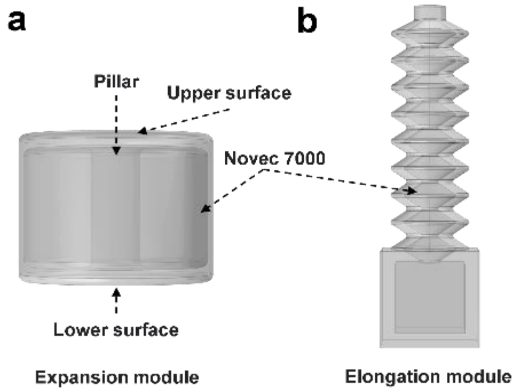

Supplementary Fig. 11: Schematic illustration showing the structure of (a) expansion actuator and (b) elongation actuator.

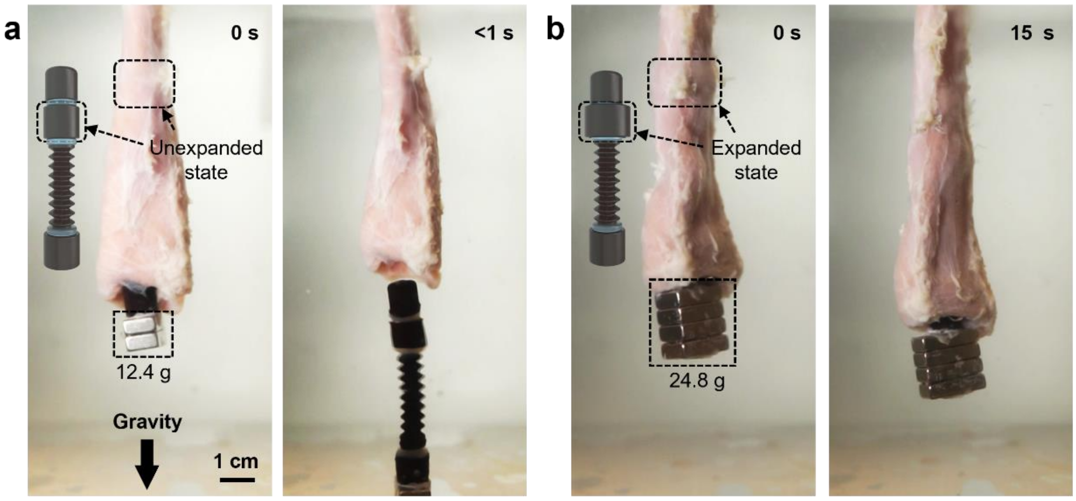

Supplementary Fig. 12: Anchoring capacity of the pipeline robot in the esophagus with loadings in (a) unexpanded and (b) expanded state. The robot's self-weight is approximately 3 g.

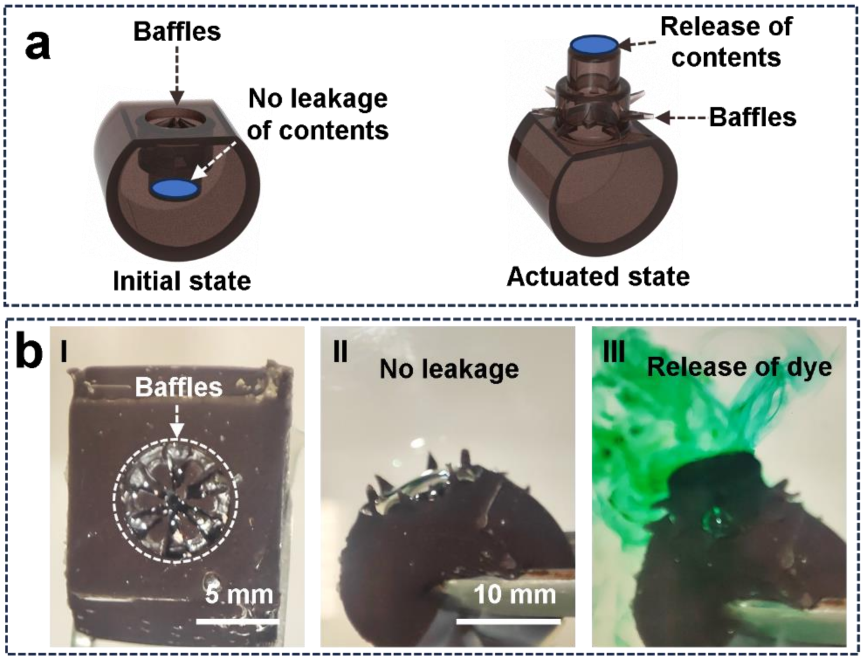

Supplementary Fig. 13: The effectiveness of the baffles in protecting the contents. (a) Schematic illustration showing the condition of the baffles before and after the device is activated. (b) Photographs showing the effectiveness of the baffles structure design to avoid the leakage.

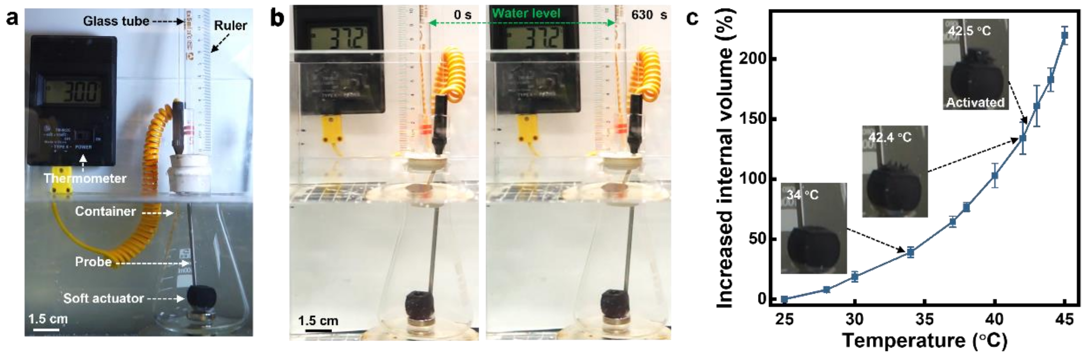

Supplementary Fig. 14: Temperature sensitivity measurement of the soft actuator. (a) Photograph of the experiment setup and (b) stability of the soft actuator in 37 °C environment. (c) The internal volume change of the soft actuator as the temperature increased. In the experiment, the initial volume of the phase-transition material inside the soft actuator was approximately 0.8 ml. When calculating the internal volume increase, the volumetric changes caused by the expansion of the water in the container were subtracted.

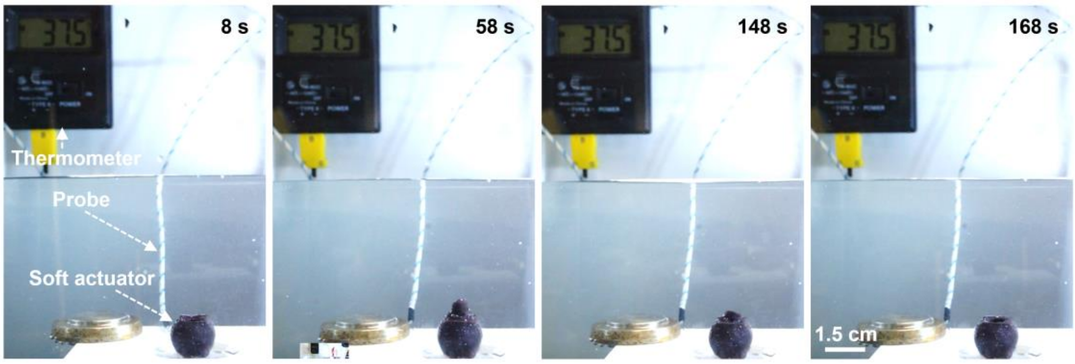

Supplementary Fig. 15: The images of the actuator extruding and retrieving the function tool actuated by ultrasound in 37.5 °C environment.

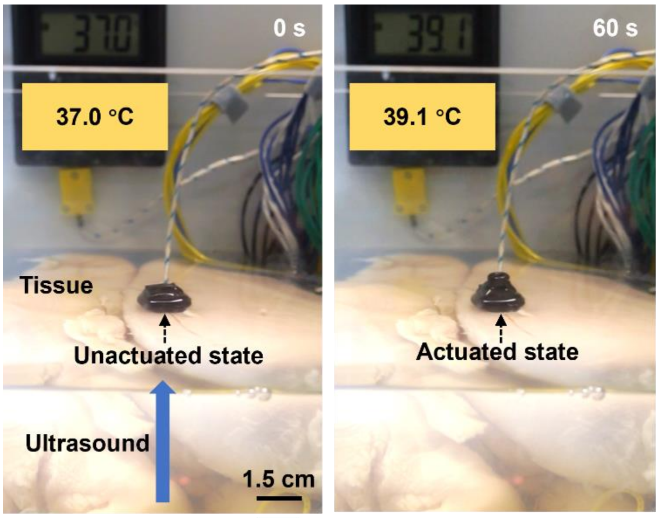

Supplementary Fig. 16: Temperature changes in tissue (in contact with the actuator) when the actuator is actuated by ultrasound waves through tissue.

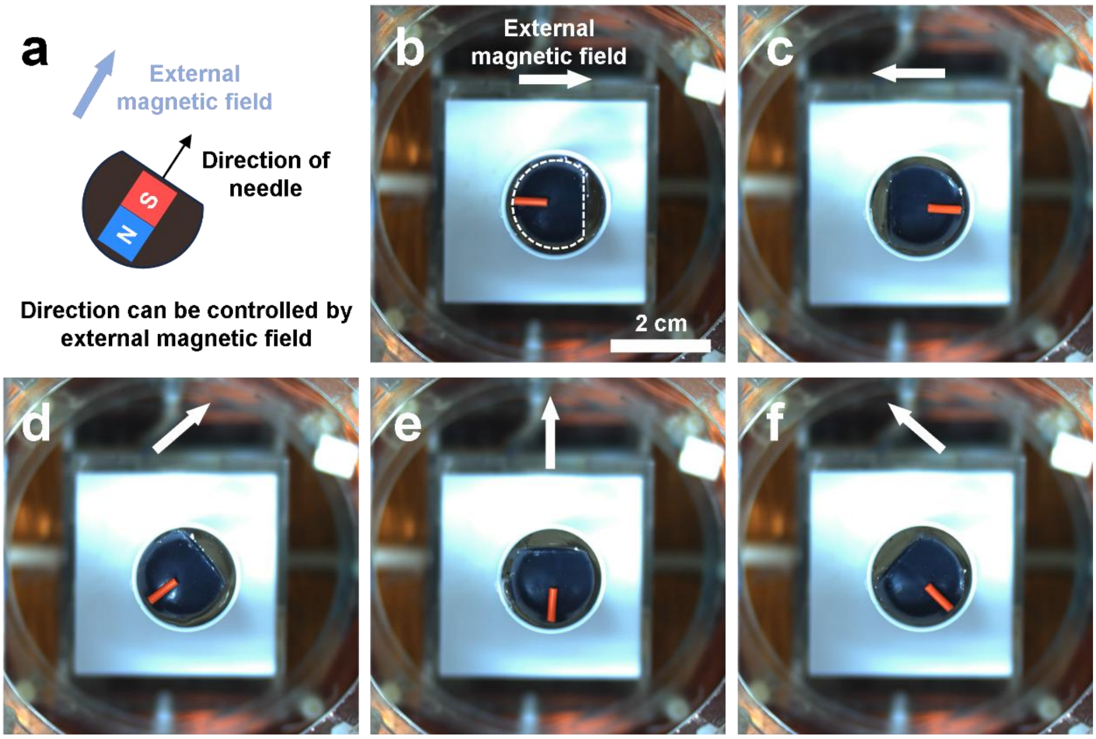

Supplementary Fig. 17: (a) Schematic illustration showing the orientation adjustment of the soft robot controlled by external magnetic field. (b) The soft robot aligns its direction with the orientation of the external magnetic field at (b) 0°, (c) 180°, (d) -45°, (e) -90° and (f) -135°, respectively.

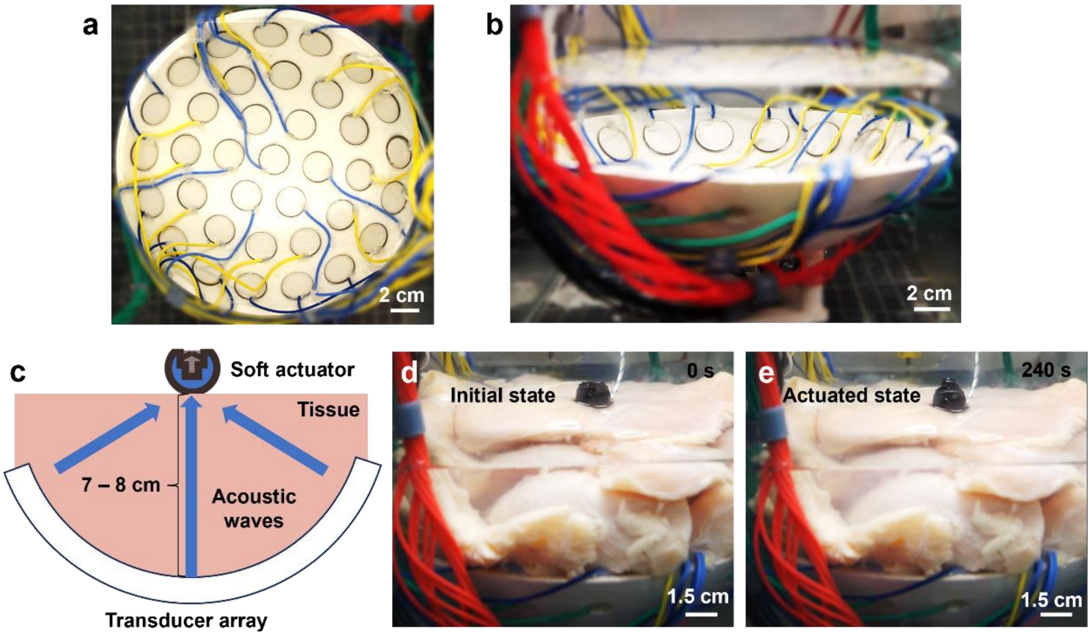

Supplementary Fig. 18: Ultrasound penetration experiment. (a) Top view and (b) side view of a focused ultrasound transducer array with 36 elements. (c) Schematic illustration of the experimental setup for assessing ultrasound penetration depth. (d) Initial state and (e) actuated state of the soft actuator before and after ultrasound excitation through tissue with the thickness of ~ 8 cm.

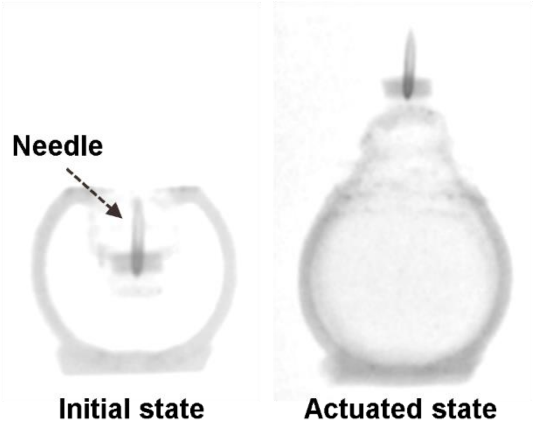

Supplementary Fig. 19: X-ray images of soft actuator for tissue acquisition in its initial and actuated state.

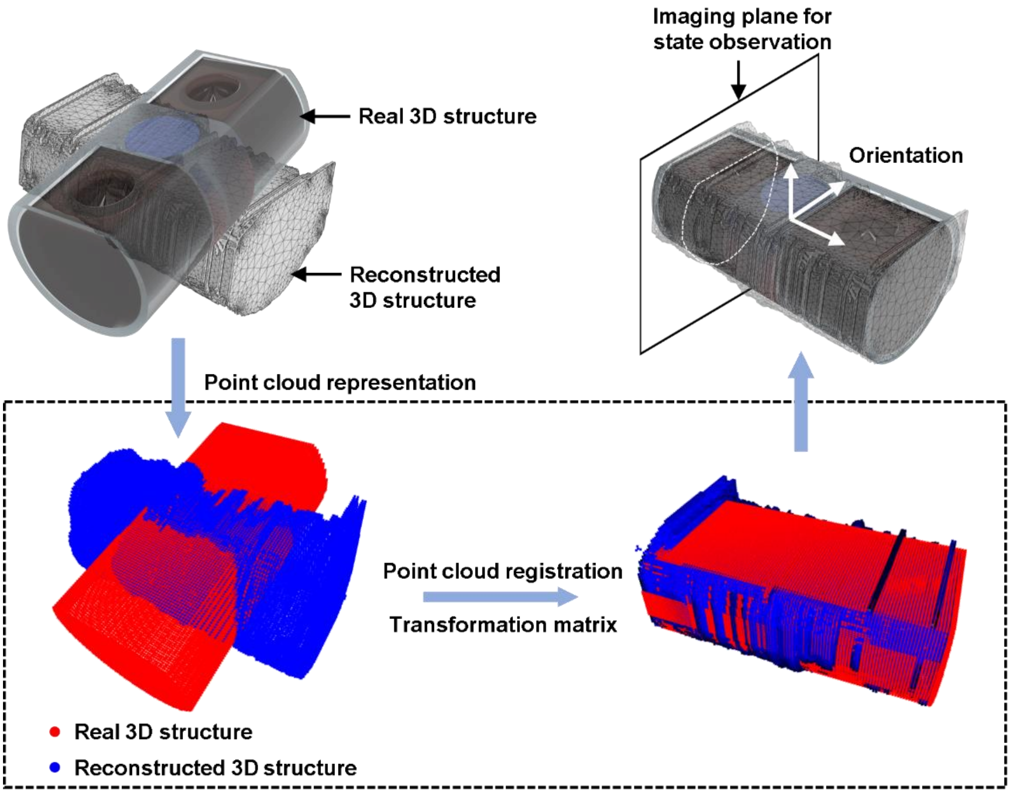

Supplementary Fig. 20: Schematic illustration of the orientation recognition method for 3D reconstructed structure.

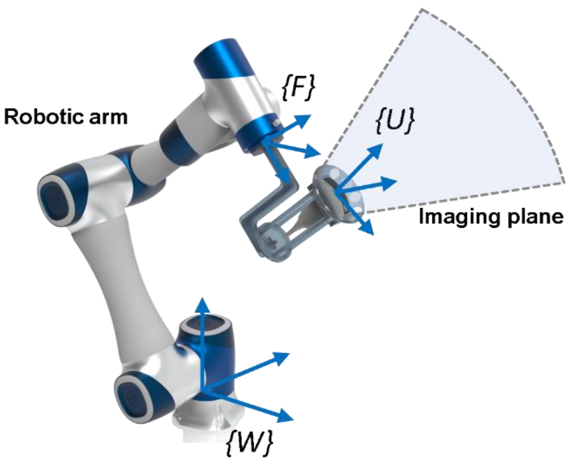

Supplementary Fig. 21: Coordinate systems, where {W}, {F}, and {U} represent world frame, robot flange frame, and ultrasound imaging plane frame, respectively.

Supplementary Table

Supplementary Table 3: The comparison of various actuation methods for untethered soft actuator and robot.

Strategy		Selective actuation	Newton-level force	Response time	Scale	Centimeter-level tissue penetration	Medical image	Ref
Quasistatic field	Magnetic soft robot	No	No (~ 60 μN)[19]	Much less than one second	Millimeter	Yes	Yes (Ultrasound)	8
		No		Much less than one second	Millimeter to centimeter	Yes	No	9
Electromagnetic wave	Liquid crystal elastomer	Yes (Spatial)	No	Less than one second	Submillimeter	No (~ 0.03-0.05 cm)[10,11]	No	12
		Yes (frequency)	No	A few seconds to tens of seconds	Millimeter to centimeter	No (~ 0.05-0.3 cm)[10,11]	No	13
	Laser-induced phase transition	Yes (Spatial)	N/A	Tens of seconds	Centimeter	No (~ 0.5 cm)[10,11]	No	14
	Millimeter wave-induced phase transition	Yes (Spatial)	Yes (38 N)	Ten seconds to tens of seconds	Centimeter	No (~ 0.05 cm)[15]	No	16
	Shape memory alloy	Yes (frequency)	No (0.6 N)	A few seconds	Millimeter to centimeter	Yes	No	17
	Shape Memory Polymer	No	No	Ten seconds to tens of seconds	Millimeter to centimeter	Yes	No	18
	Coiled artificial muscle	No	Yes (~3.1 N)	A few seconds	Millimeter to centimeter	Yes	No	19
	Radio frequency-induced phase transition	No	Yes (3.1 N to 70 N)	A few seconds	Millimeter	Yes	Yes (Ultrasound)	20
		No	Yes (up to 31 N)	Ten seconds to tens of seconds	Centimeter	Yes	No	21
**Mechanical wave**	**Focused ultrasound-controlled phase transition**	**Yes (Spatial)**	**Yes (up to 5.5 N)**	**A few seconds to tens of seconds**	**Millimeters to centimeter**	**Yes (~ 8 cm)**	**Yes (Ultrasound, X-ray)**	**This work**

Supplementary Reference

1. Hughes, S. Medical Ultrasound Imaging. *Phys. Educ.* **36**, 468 (2001).
2. Martínez, R., Vera, A. & Leija, L. HIFU Induced Heating Modelling by Using the Finite Element Method. *Phys. Procedia* **63**, 127–133 (2015).
3. Venzac, B. *et al.* PDMS Curing Inhibition on 3D-Printed Molds: Why? Also, How to Avoid It? *Anal. Chem.* **93**, 7180–7187 (2021).
4. Xavier, M. S., Fleming, A. J. & Yong, Y. K. Finite Element Modeling of Soft Fluidic Actuators: Overview and Recent Developments. *Adv. Intell. Syst* **3**, 2000187 (2021).
5. Holzapfel, G. A. Nonlinear Solid Mechanics: A Continuum Approach for Engineering Science. *Meccanica* **37**, 489–490 (2002).
6. Ogden, R. W. Large Deformation Isotropic Elasticity - On the Correlation of Theory and Experiment for Incompressible Rubberlike Solids. *Proc. Math. Phys. Eng. Sci. P ROY SOC A-MATH PHY* **326**, 565–584 (1972).
7. Steck, D. *et al.* Mechanical Responses of Ecoflex Silicone Rubber: Compressible and Incompressible Behaviors. *J. Appl. Polym. Sci.* **136**, 47025 (2019).
8. Hu, W., Lum, G. Z., Mastrangeli, M. & Sitti, M. Small-scale Soft-bodied Robot with Multimodal Locomotion. *Nature* **554**, 81–85 (2018).
9. Dong, Y. *et al.* Untethered Small-scale Magnetic Soft Robot with Programmable Magnetization and Integrated Multifunctional Modules. *Sci. Adv.* **8**, eabn8932 (2022).
10. Bashkatov, A. N., Genina, E. A., Kochubey, V. I. & Tuchin, V. V. Optical properties of human skin, subcutaneous and mucous tissues in the wavelength range from 400 to 2000 nm. *J. Phys. D: Appl. Phys.* **38**, 2543 (2005).

11. Avci, P. *et al.* Low-level Laser (Light) Therapy (LLLT) in Skin: Stimulating, Healing, Restoring. *Semin. Cutan. Med. Surg.* **32**, 41–52 (2013).

12. Palagi, S. *et al.* Structured Light Enables Biomimetic Swimming and Versatile Locomotion of Photoresponsive Soft Microrobots. *Nat. Mater* **15**, 647–653 (2016).

13. Zuo, B., Wang, M., Lin, B.-P. & Yang, H. Visible and Infrared Three-wavelength Modulated Multi-directional Actuators. *Nat. Commun.* **10**, 4539 (2019).

14. Meder, F., Naselli, G. A., Sadeghi, A. & Mazzolai, B. Remotely Light-Powered Soft Fluidic Actuators Based on Plasmonic-Driven Phase Transitions in Elastic Constraint. *Adv. Mater.* **31**, 1905671 (2019).

15. Wu, T., Rappaport, T. S. & Collins, C. M. The Human Body and Millimeter-wave Wireless Communication Systems: Interactions and Implications. in *2015 IEEE Int. Conf. Commun. (ICC)* 2423–2429 (2015).

16. Ueno, S. & Monnai, Y. Wireless Soft Actuator Based on Liquid-Gas Phase Transition Controlled by Millimeter-Wave Irradiation. *IEEE Robot. Autom. Lett.* **5**, 6483–6488 (2020).

17. Boyvat, M., Koh, J.-S. & Wood, R. J. Addressable wireless actuation for multijoint folding robots and devices. *Sci. Robot.* **2**, eaan1544 (2017).

18. Ze, Q. *et al.* Magnetic Shape Memory Polymers with Integrated Multifunctional Shape Manipulation. *Advanced Materials* **32**, 1906657 (2020).

19. Li, M. *et al.* Miniature Coiled Artificial Muscle for Wireless Soft Medical Devices. *Sci. Adv.* **8**, eabm5616 (2022).

20. Tang, Y. *et al.* Wireless Miniature Magnetic Phase-Change Soft Actuators. *Adv. Mater.* **34**, 2204185 (2022).

21. Mirvakili, S. M., Sim, D., Hunter, I. W. & Langer, R. Actuation of Untethered Pneumatic Artificial Muscles and Soft Robots Using Magnetically Induced Liquid-to-gas Phase Transitions. *Sci. Robot.* **5**, eaaz4239 (2020).
