# Supplementary material for: Focused ultrasound enables selective actuation and Newton-level force output of untethered soft robots
Source: Nat Commun. 2024 Jun 18;15:5197. doi: 10.1038/s41467-024-49148-6 (PMC11189400; doi:10.1038/s41467-024-49148-6)
Supplement: Supplementary file 3 — Description of Additional Supplementary Files [file 41467_2024_49148_MOESM3_ESM.pdf]

<u>Description of Additional Supplementary Information Files</u>

Supplementary Movie 1. Ultrasound-powered expansion and elongation actuators and in-pipe soft robot.

Supplementary Movie 2. FUPT-based soft robot for selective release and on-demand delivery of liquid cargo.

Supplementary Movie 3. Ultrasound-powered soft robot for biopsy and tissue patching.

Supplementary Movie 4. Imaging and control system for FUPT actuated soft robot.
